# Exploring the impact of efficacy messages on cessation-related outcomes using Ecological Momentary Assessment

**DOI:** 10.18332/tid/94460

**Published:** 2018-09-21

**Authors:** Victoria Lambert, Stuart G. Ferguson, Jeff Niederdeppe, David Hammond, James W. Hardin, James F. Thrasher

**Affiliations:** 1 Department of Health Promotion, Education, & Behavior, Arnold School of Public Health, University of South Carolina, Columbia, United States; 2 School of Medicine, University of Tasmania, Hobart, Australia; 3 Department of Communication, Cornell University, Ithaca, United States; 4 School of Public Health & Health Systems, University of Waterloo, Waterloo, Canada; 5 Department of Epidemiology & Biostatistics, Arnold School of Public Health, University of South Carolina, Columbia, United States; 6 Department of Tobacco Research, Center for Population Health Studies, National Institute of Public Health, Cuernavaca, Mexico

**Keywords:** smoking cessation, health policy, tobacco control, health communication

## Abstract

**INTRODUCTION:**

Observational studies indicate that cigarette package inserts with efficacy messages about the benefits of quitting (i.e. response efficacy) and recommendations for successful cessation increase smokers’ self-efficacy to quit and promote sustained cessation. However, the effects of inserts with such efficacy messages have not been studied using experimental designs. This study used ecological momentary assessment (EMA) to assess smokers’ responses to efficacy inserts.

**METHODS:**

In a randomized case-crossover study among smokers from the United States (n=23), participants received a one-week supply of cigarettes with efficacy inserts and a one-week supply without any inserts, and were randomized to use the packs with inserts on either the first or second week of the study. For 14 consecutive days, participants used a smartphone to answer brief surveys on cessation-related variables during smoking sessions and at the beginning of each day. Multilevel mixed-effects linear and logistic regression models compared responses during the insert period to those of the non-insert period.

**RESULTS:**

The insert period was associated with greater desire to quit (b=0.21, p=0.012), motivation to quit (b=0.18, p=0.001), self-efficacy to cut down (b=0.26, p<0.001) and to quit (b=0.28, p<0.000), and response efficacy/perceived benefits of quitting (b=0.13, p=0.012). Insert exposure was not significantly associated with forgoing cigarettes (OR=1.9, p=0.2)

**CONCLUSIONS:**

Results from this EMA study suggest that inserts with efficacy messages may promote determinants of smoking cessation. This is consistent with observational research in Canada, which is the only country to use inserts with efficacy messages as well as pictorial warnings about smoking risks on the outside of packs. Future studies should assess the extent to which efficacy inserts can not only be used to communicate health information to smokers but also work in synergy with pictorial warnings.

## INTRODUCTION

While many countries and jurisdictions have implemented pictorial health warning labels (HWLs) on the outside of cigarette packs, Canada is the only country that also requires complementary messaging on inserts inside the packs. Canadian inserts include supportive health messages on the benefits of quitting (i.e. response efficacy messages) and recommendations for successful smoking cessation (i.e. self-efficacy messages). Observational studies suggest that these inserts increase smokers’ self-efficacy to quit^1^ and promote quit attempts and sustained smoking cessation^1,2^. Further, experimental studies indicate that smokers perceive inserts with Canadian-style efficacy messages to be motivating and useful for quitting^3,4^. The current study assessed the real-world effects of inserts on cessation-related variables among a sample of smokers from the United States.

The cessation-related effects of pictorial HWLs that highlight smoking risks using graphic imagery appear to be partially due to increased negative affect, including fear^5^. As suggested by fear-appeal research and theories^6,7^, a combination of self-efficacy and response efficacy messages can enhance fear-appeal messages and promote desirable attitudinal and behavioral change independent of fear arousal. However, the effects of inserts with efficacy messages have not been assessed in experiments outside brief, one-time exposures to messages online^3,4^. Further research is needed to determine if these supportive messages are effective.

This study used ecological momentary assessment (EMA) methods to assess smokers’ real-time responses to inserts with efficacy messages in the US, where warning labels are small and do not include pictures. EMA has been used in observational studies to assess smokers’ responses to pictorial HWLs with fear-arousing content^8-10^, but not to assess responses to efficacy messages on inserts. While the primary purpose of this study was to test the feasibility of the study procedures, we expected that the period of exposure to efficacy inserts would be associated with greater self-efficacy to reduce and completely quit smoking, response efficacy, motivation and desire to quit, and forgoing cigarettes, when compared to the period without exposure to efficacy inserts.

## METHODS

### Sample

Data collection occurred between 19 July 2017 and 11 February 2018, in South Carolina and New York. Smokers in South Carolina were recruited through e-mail, Facebook, Craigslist, and Instagram advertisements. Smokers in New York were recruited through word-of-mouth and street intercepts outside tobacco shops. Potential participants completed an online survey to assess eligibility, which included: smoking at least 100 cigarettes in their lifetime; being a daily smoker; smoking 10 or more cigarettes per day; being 18–50 years of age; planning to quit sometime in the future; and no use of e-cigarettes or any other combustible/smokeless tobacco product in the prior month.

### Study protocol

EMA involves using mobile devices to query participants at critical moments, such as during smoking sessions when people are exposed to messages on and in cigarette packages, potentially mitigating recall bias^11^. For this study, EMA data were collected using smartphones programmed with a customized app installed and other device functions disabled (http://www.utas.edu.au/health/research/groups/behavioural-and-situational-research-group-bsrg/hbart).

Participants attended a pre-study orientation that involved: confirmation of smoking status using a carbon monoxide breathalyzer (minimum 8 ppm CO required); a brief survey; and training in the study protocol. Participants were given a two-week supply of their preferred cigarette brand variety: one week’s supply with inserts and one week’s supply without inserts. Participants were randomized to use the supply with inserts on either the first or second week of the study. Packs with inserts contained one of four different insert messages, and participants received at least one of each of the four messages in their supply. Of the four insert messages, two contained response efficacy information and two contained self-efficacy information, all of which included the phone number for a smoking cessation quit-line and website (smokefree.gov; see Supplementary Appendix). Each week’s supply was labeled as ‘Set 1’ or ‘Set 2’ and placed in separate, zip-locked bags labeled with the days on which participants should use the packs.

Participants used the smartphone provided to complete a survey each morning and to indicate each time they smoked a cigarette during the 14 consecutive days of the study period. The morning survey queried how long ago the participant had awaken and if they had stubbed out a cigarette early or not smoked when they intended to do so in the prior 24 hours (i.e. forgoing). As a manipulation check of insert exposure, the morning log also prompted participants to take a photo of their cigarette pack.

Participants could log cigarettes at any time of day, and, when they logged a cigarette, the app queried whether they were smoking and if the current cigarette was from a new pack. Participants were prompted to complete a brief survey (i.e. event-related survey) every time they opened a new pack and, on average, at three additional, randomly selected smoking events each day (the algorithm considered the baseline quantity of cigarettes smoked per day in determining the frequency of surveys). Event-related surveys queried feelings about smoking, desire to quit smoking, motivation to quit smoking, self-efficacy to reduce smoking, self-efficacy to quit smoking, response efficacy (i.e. perceived benefits of quitting), and worry about the effects of smoking (see Table 1 for question wording). After the 14-day EMA period ended, participants attended a de-briefing session, during which they completed an online survey on perceptions of the insert messages, participated in a qualitative interview to discuss any issues completing the study protocol, and received US$150 in remuneration, along with a pamphlet containing information on free smoking-cessation resources.

**Table 1:** Results for key outcomes, comparing the period with exposure versus without exposure to inserts with efficacy messages^a^

*Variable*	*Item*	*Response format and options (slider)*	*Coef.*	*(SE)*
Feeling toward smoking	Right now, you feel like smoking is…?	1 = very bad to 7 = very good	-0.10	0.06
Desire to quit	How strong is your desire to quit at this time?	1= not at all strong to 7= extremely strong	0.21*	0.08
Motivation to quit	How motivated are you to quit smoking?	1= not at all to 7= extremely	0.18***	0.06
Self-efficacy to cut down	How easy would it be to cut down on the number of cigarettes you smoke?	1 = not at all easy to 9 = extremely easy	0.26***	0.08
Self-efficacy to quit	How confident are you that you could quit smoking altogether right now?	1 = not at all confident to 7 = extremely confident	0.28***	0.06
Response efficacy	How much would quitting smoking now reduce your chances of getting a serious disease?	1 = no chance to 7 = certain to happen	0.13*	0.05
Worry about the risks of smoking	How worried are you about the possible effects of smoking?	1 = not at all worried to 7 = extremely worried	-0.07	0.07
Forgoing cigarettes (odds ratio)^b^	In the last 24 hours, have you stubbed out a cigarette early or not had a cigarette when you would normally?	Yes/No	1.9	0.90

a Models were estimated separately for each outcome and adjusted for multiple responses by each individual as well as the week in which participants were exposed to inserts (i.e. 1st vs 2nd week of study). Models include data from 23 participants. b Forgoing was assessed once each day. All other outcomes in Table 1 were assessed multiple times each day, around smoking events.

* p<0.05, **p<0.01

*** p<0.001.

### Analyses

Multilevel mixed-effects linear and logistic models regressed outcomes from event-related surveys and morning reports on an indicator variable for the period when participants were exposed versus not exposed to inserts, controlling for week of insert exposure (i.e. 1st vs 2nd week). For outcomes that were measured multiple times per day (i.e. event-related surveys), three-level models were estimated, considering observations as nested within days and within participants. For the forgoing outcome, which was measured once daily, two-level models considered observations nested within participants only. Stata 15 was used for all analyses (StataCorp. 2017. Stata Statistical Software: Release 15. College Station, TX: StataCorp LLC).

## RESULTS

### Compliance and descriptive findings

Participants reported no serious issues with completing the protocol. Of the 28 participants who began the study, 27 completed it. Four of these 27 participants were excluded from analysis because information acquired from the follow-up interview and the manipulation checks indicated they were not exposed to the inserts as assigned. Three participants did not use the insert and non-insert packs as assigned, and one participant indicated that he never saw the inserts, which was likely because they were hidden due to the type of pack for his preferred brand.

For the analytic sample of 23 smokers, the mean age was 36.2 years, and about half (52%) was female, half intended to quit within the next 6 months (48%), and a third (35%) had attempted to quit in the prior 4 months. The mean Heaviness of Smoking Index^12^ was 3.41 (SE=0.22). The total number of cigarettes logged was 2744 (with a mean, M=8.86 per day during insert period, and M=9.08 per day during non-insert period). On average, participants completed 13.7 (of 14) days of the study protocol, 40.6 event-related surveys, and 10.4 (of 14) morning reports throughout the study period. Compliance with the event-related surveys ranged from 88 to 100%. The average number of days exposed to inserts was 6.8 (of 7).

### Insert vs non-insert period

Table 1 shows the results from the mixed-effects regression models, comparing observations during the period when participants were exposed to inserts and when they were not. The insert period was associated with greater desire to quit (b=0.21, SE=0.08, p=0.01), stronger motivation to quit (b=0.18, SE=0.06, p=0.001), stronger self-efficacy to cut down (b=0.26, SE=0.08, p=0.001) and to quit (b=0.28, SE=0.06, p<0.000), and greater response efficacy (b=0.13, SE=0.05, p=0.01). Insert exposure was not significantly associated with feeling toward smoking (b=-0.10 SE=0.06, p=0.10), worry about the effects of smoking (b=-0.07, SE=0.07, p=0.33) or forgoing cigarettes (OR=1.9, SE=0.9, p=0.2). The order of insert exposure (i.e. 1st vs 2nd week) was not significantly associated with any outcome (results not shown).

## DISCUSSION

The EMA protocol used in this study appears acceptable and feasible. While the findings should be confirmed in a larger study, five of the six study hypotheses regarding the positive effects of insert exposure on determinants of smoking cessation were supported. The findings that insert exposure was positively associated with self-efficacy to cut down and quit are particularly important since there is ample evidence that increasing self-efficacy promotes smoking cessation^13-16^.

Contrary to our hypothesized expectation, we did not find a statistically significant association between insert exposure and forgoing cigarettes, although the coefficient suggested a tendency towards more forgoing when exposed to inserts. We had relatively low power to detect this effect because data on forgoing were only collected once a day compared to multiple times a day for the other study outcomes. Larger sample sizes and longer follow-up are likely necessary to better assess forgoing cigarettes, which consistently predicts quit attempts^17-19^. Overall, our results suggest that inserts have medium-sized effects^20^ on determinants of cessation; however, these effects may partly reflect demand-effects from our case-crossover design, which did not blind participants to the experimental period. Between-subject study designs would better control for this potential bias but would likely require larger sample sizes.

Our study had some additional limitations, including the inability to assess cessation behavior over a short period of time. Similarly, it is also unclear how long insert effects would be sustained given evidence^21,22^ of ‘wear out’ for HWLs; nevertheless, observational research in Canada found that attention to inserts increased over time, even while attention to HWLs dissipated^1^. More research is needed to confirm study findings and better inform cigarette labeling policy. Future studies should assess potentially synergistic effects of inserts and HWLs, since theory suggests that efficacy messages can enhance the effects of fear-arousing messages^7^ and HWL research suggests that fear arousal might promote more deliberate processing of labeling messages^23^. Furthermore, although theory emphasizes the importance of both response efficacy and self-efficacy messages, future research could investigate this premise by assessing their independent effects on cessation-related outcomes.

## CONCLUSIONS

The preliminary findings from this pilot study suggest that efficacy messages promote determinants of cessation. These findings warrant further assessment in larger trials that randomize participants to different labeling conditions, including assessment of potential synergies between efficacy messages and fear-arousing messages.

## CONFLICTS OF INTEREST

S. G. Ferguson has consulted for GlaxoSmithKline Consumer Healthcare and Chrono Therapeutics Inc. on matters relating to smoking cessation, and has received researcher-initiated project grant funding (through the GRAND initiative) and travel funds from Pfizer. He has also served on an advisory board for Johnson & Johnson. D. Hammond has provided paid expert witness testimony in legal proceedings on behalf of governments, including tobacco industry challenges to health warning regulations, outside the submitted work. The rest of the authors have also completed and submitted an ICMJE form for disclosure of potential conflicts of interest. The authors declare that they have no competing interests, financial or otherwise, related to the current work.
