# Supplementary material for: Exploring the impact of efficacy messages on cessation-related outcomes using Ecological Momentary Assessment
Source: Tob Induc Dis. 2018 Sep 21;16:44. doi: 10.18332/tid/94460 (PMC6659513; doi:10.18332/tid/94460)
Supplement: Supplementary file 1 [file TID-16-44-s1.pdf]

**Supplementary appendix: Efficacy inserts used in the EMA pilot study**

## Self-efficacy inserts

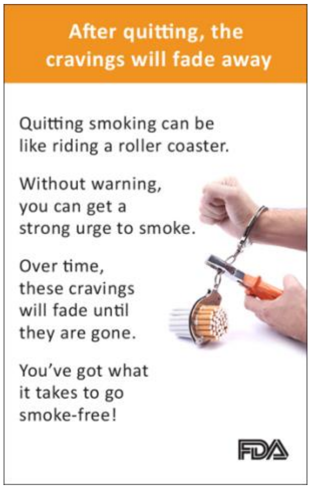


Self-efficacy message 1- Front

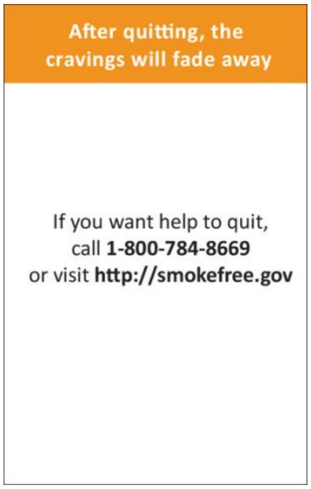


Self-efficacy message 1- Back

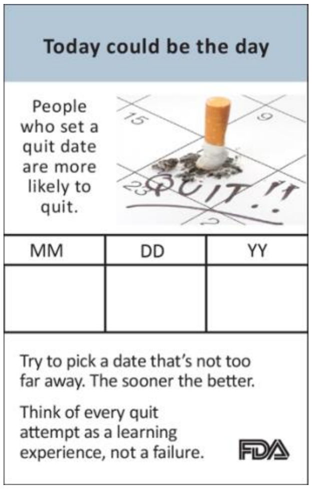


Self-efficacy message 2- Front

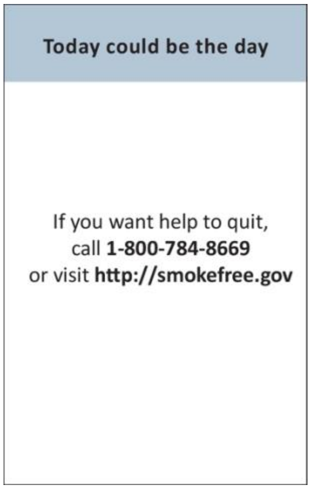


Self-efficacy message 2- Back

## Response efficacy inserts

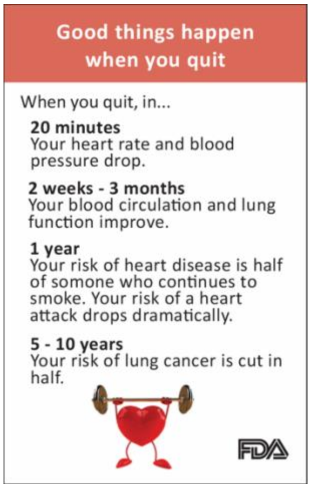


Response efficacy message 1- Front

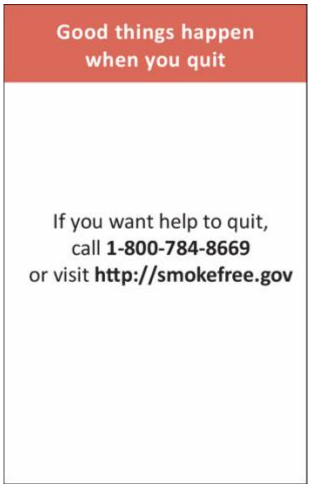


Response efficacy message 1- Back

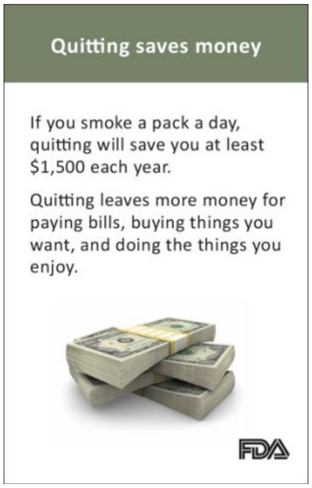


Response efficacy message 2- Front

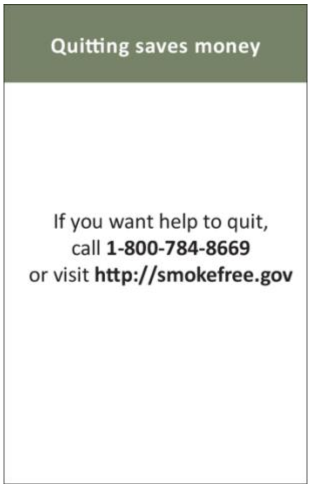


Response efficacy message 2- Back

© 2018 Lambert V.
